# Human immunodeficiency virus-negative plasmablastic lymphoma in the neck: a rare case report and literature review

**DOI:** 10.1186/s40001-014-0064-6

**Published:** 2014-11-22

**Authors:** Pengli Jiang, Min Liu, Bailong Liu, Bin Liu, Yuhua Zhou, Lihua Dong

**Affiliations:** Department of Breast Surgery, The First Hospital, Jilin University, 71 Xinmin Street, Changchun, 130021 China; Department of Radiation Oncology, The First Hospital, Jilin University, 71 Xinmin Street, Changchun, 130021 China; Department of Hand Surgery, The First Hospital, Jilin University, 71 Xinmin Street, Changchun, 130021 China; Department of Pathology, The First Hospital, Jilin University, 71 Xinmin Street, Changchun, 130021 China

**Keywords:** HIV-negative, Plasmablastic lymphoma, Radiotherapy

## Abstract

Plasmablastic lymphoma (PBL) is an aggressive neoplasm exclusively occurring in AIDS patients. Recently, increasing cases of human immunodeficiency virus (HIV)-negative PBL have been reported. No standard therapy protocol is currently available since there is a great difference between PBL with and without HIV infection. Here, we present a rather rare case of HIV-negative PBL in the neck that dramatically responded to radiotherapy alone. Our case highlights the possibility of PBL in the neck and helps to expand our understanding of this separate lymphoma. The related literature review summarized the clinicopathological features and treatment status of HIV-negative PBL.

## Background

Plasmablastic lymphoma (PBL) is a rare entity which mostly involves the oral cavity of human immunodeficiency virus (HIV)-positive individuals. Recently, increasing cases have been reported in HIV-negative patients [1-5]. Extraoral sites, such as the central nervous system, maxillary sinus, nasal cavity, gastrointestinal tract, liver, and retroperitoneal region, can also be involved [1-4,6-9]. Many differences on the aspect of the clinicopathologic features and treatment response between HIV-positive and -negative cases have been revealed [10]. Here, we present a PBL case in an immunocompetent woman for which radiotherapy alone provided a remarkable response without severe side effects or intensive chemotherapy.

## Case presentation

A 76-year-old female presented on October 22^nd^, 2013, with a history of a painless lump in the left region of the neck for about a month. She also complained of hoarseness and drinking cough for one week. She did not suffer from fever, night sweat, or significant weight loss. Her past history was unremarkable except for hypertension for about 5 years without regular treatment and allergy to penicillin and cephalosporin. She denied any prior immunosuppressive conditions.

On physical examination, her blood pressure was 146/70 mmHg. The trachea shifted to the right side. A 5 × 4 cm firm mass was palpated in the lower left neck. No enlarged lymph nodes were palpable in the bilateral axilla and groin. Sternum tenderness was negative.

Laboratory investigations demonstrated a normal complete blood cell count and a generally normal serum biochemical profile. The levels of IgG, IgA, IgM, and lactate dehydrogenase were normal. Erythrocyte sedimentation rate was elevated at 45 mm/h (normal, <20 mm/h). β2-microglobulin was mildly elevated at 2.78 mg/L (normal, <1.8 mg/L). Serum and urinary immunofixation electrophoresis were both negative. The content of κ and λ free light chain in the 24 h urine was normal. The level of hepatitis B antigen was normal. Serum antibodies for hepatitis C, HIV, and syphilis were negative.

**Figure 1:**
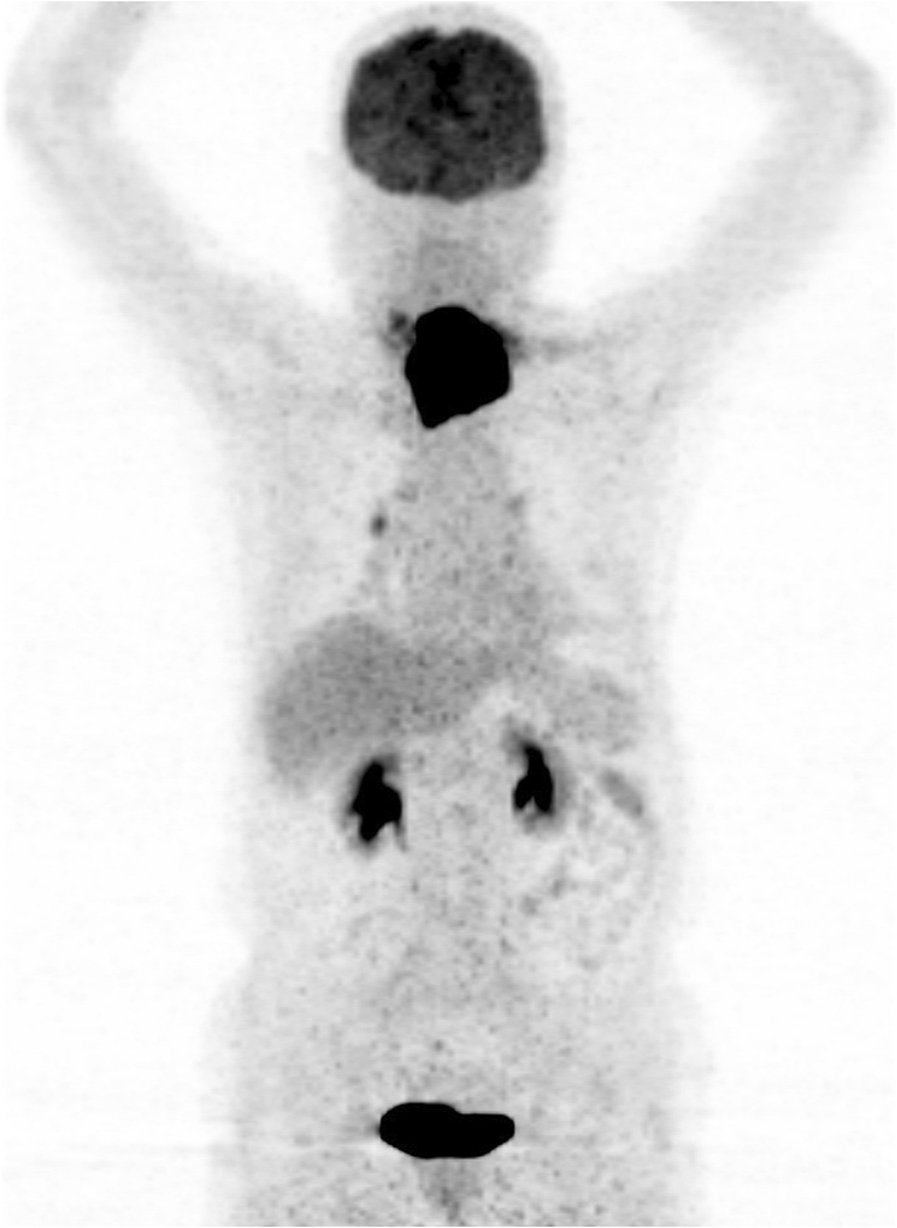
**Whole body PET/CT scan evaluated the involvement.** A hypermetabolic mass in the left supraclavicular fossa involved the left lobe of the thyroid. The large primary tumor compressed adjacent organs such as oropharynx, hypopharynx, larynx, trachea, and esophagus. No other abnormal FDG uptake lesions were found.

**Figure 2:**
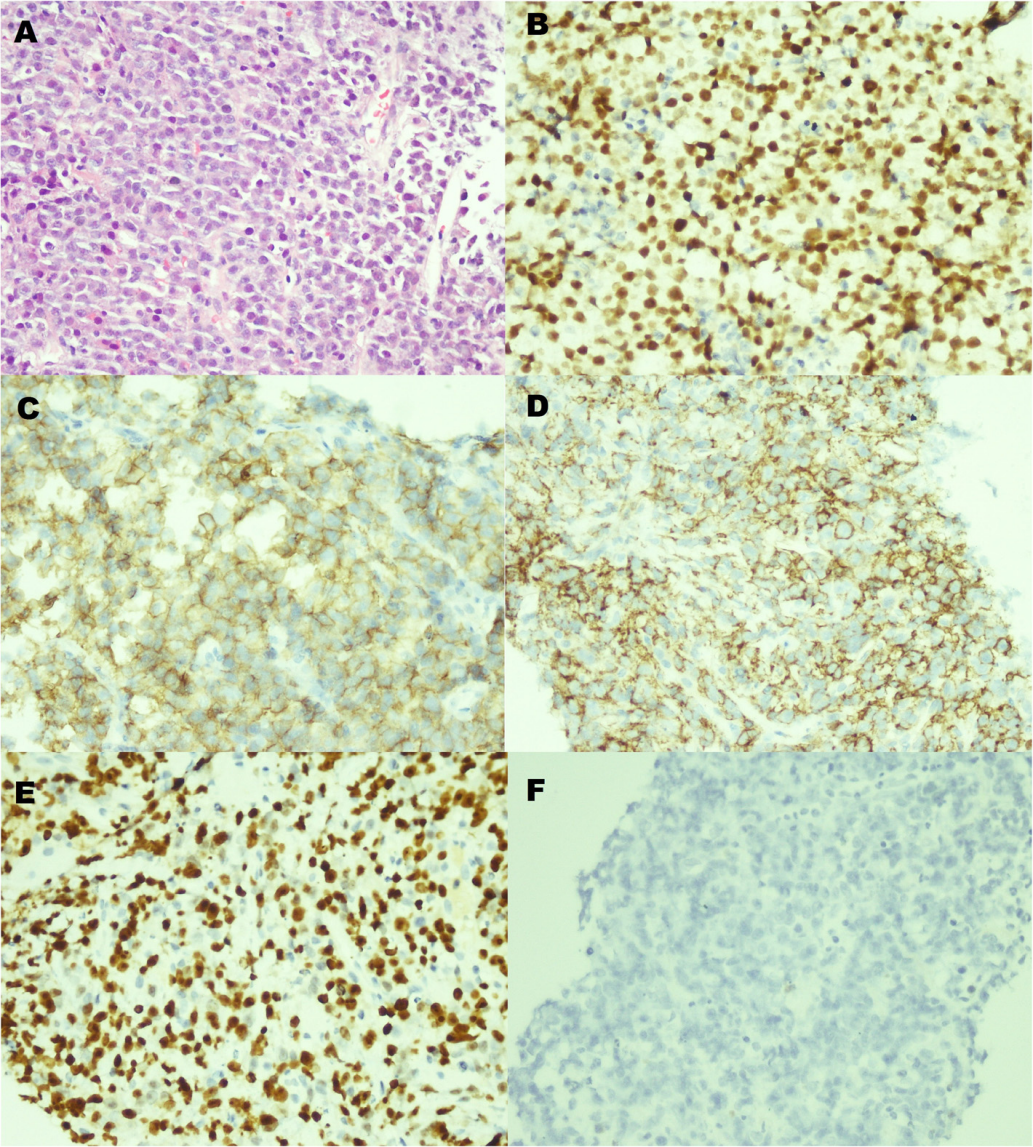
**Histopathologic results of biopsy specimen of the left supraclavicular mass. (A)** Hematoxylin-eosin stained section (×40) revealed that diffuse infiltration of medium to large atypical cells with round nuclei and prominent nucleolus. **(B–D)** Immunochemistry examination for Mum-1, CD 38, and CD 138 were intensively positive for neoplastic cells (×40). **(E)** Immunochemistry showed Ki-67 expressed in the nuclei of 80% of neoplastic cells (×40). **(F)** In situ hybridization revealed negativity for Epstein-Barr virus-encoded small RNA (EBER) (×40).

Laryngoscopy showed left vocal cord paralysis. Ultrasound of the neck found a 50 mm × 44 mm heterogeneous hypoechoic mass in the left supraclavicular fossa which had no clear delineation with the left lobe of the thyroid. The patient then underwent a whole body PET/CT scan which demonstrated a hypermetabolic mass in the left supraclavicular fossa involving the left lobe of the thyroid (Figure 1). There was no other abnormal FDG uptake lesion. Biopsy of the lump revealed diffuse infiltration of medium to large atypical cells with round nuclei and prominent nucleolus (Figure 2A). Neoplastic cells strongly expressed Mum-1 (Figure 2B), CD 38 (Figure 2C), CD 138 (Figure 2D), and Ki-67 (80% expression, Figure 2E). The immunochemistry showed CK(–), CD3(–), CD7(–), LCA(+), CD20(–), pax-5(–), CD30(–), TdT(–), and CD34(–). In situ hybridization revealed negativity for Epstein-Barr virus-encoded small RNA (EBER) (Figure 2F). Bone marrow aspiration did not reveal bone marrow involvement. Thus, the patient was diagnosed as PBL IA with an International Prognostic Index score of 1.

**Figure 3:**
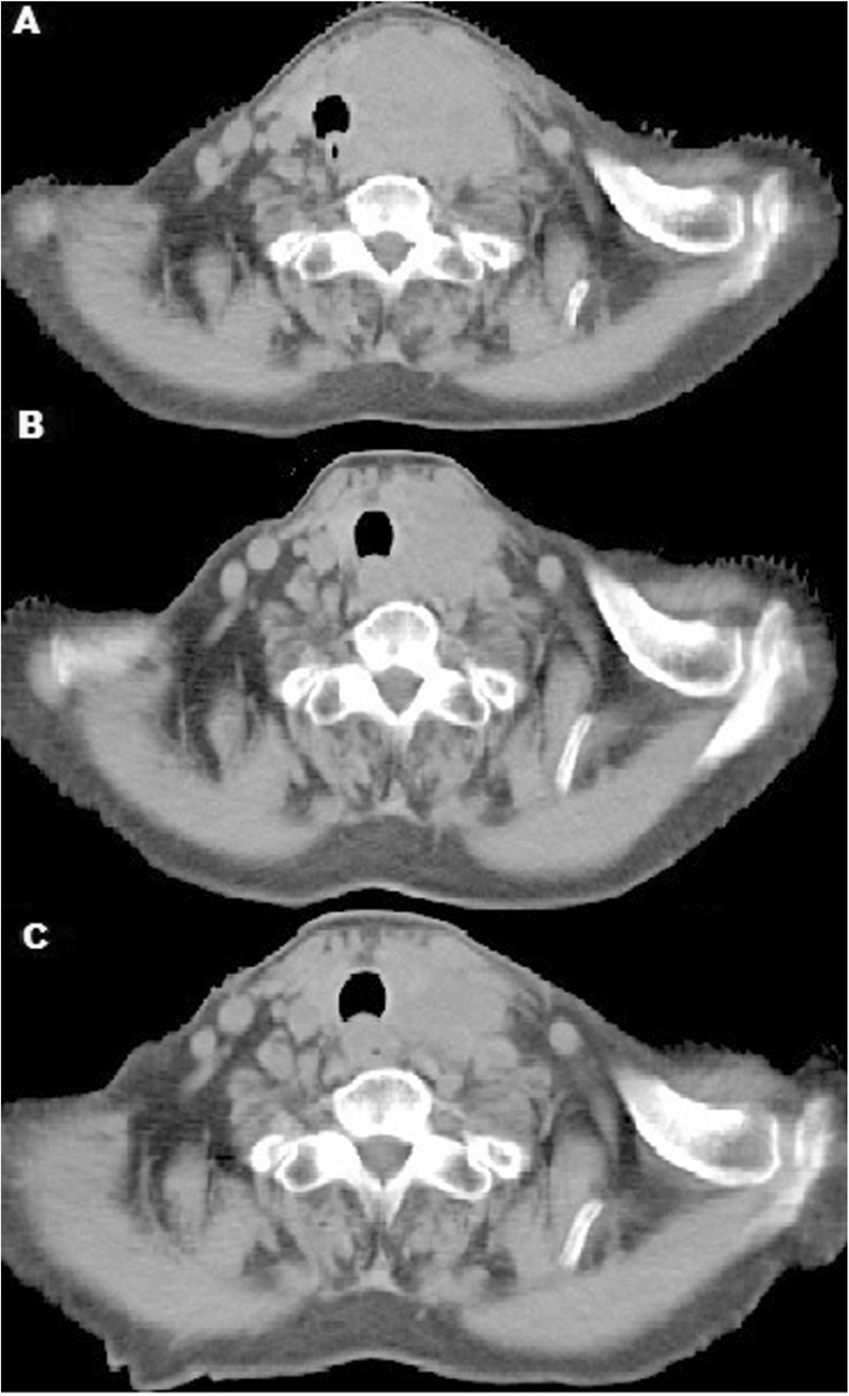
**Effect evaluation between pre-radiotherapy, radiotherapy with a dose of 28 Gy/14 f, and after radiotherapy of 56 Gy/28 f. (A)** Before radiotherapy the giant primary tumor compressed the trachea and esophagus and invaded the left lobe of the thyroid. **(B)** The tumor shrank greatly after irradiation of 28 Gy/14 f. **(C)** After radiotherapy of 56 Gy/28 f, the tumor was minimal residual and the response was near complete remission.

Because of the patient’s age, intensive chemotherapy was not performed. Instead, a vincristine and prednisone pretreatment regimen was given followed by 300 mg cyclophosphamide and 10 mg dexamethasone intravenously. The tumor had no response. However, the patient suffered from fungal infection. Due to the severe side effect, chemotherapy ceased and an effective antifungal treatment was administered along with best supportive care. In order to rapidly relieve the symptoms, she underwent radiation to the primary tumor by intensity-modulated radiotherapy (IMRT). After a dose of 28 Gy/14 f, the tumor shrank significantly with a 71.4% response and all the discomforts disappeared (Figure 3A,B). The radiotherapy plan was then adjusted and the lymphatic drainage area of the bilateral neck and the residual tumor were irradiated at a dose of 28 of Gy/14 f by the RapidArc® technique. After radiation with a dose of 56 Gy/28 f, the tumor became smaller and the lesion exhibited an 87% response (Figure 3C). To date, the patient is still alive.

### Discussion

PBL was first described in the oral cavity of HIV-infected individuals in 1997 by Delecluse et al. [11]. It was considered to have a strong correlation with HIV infection, accounting for about 2.6% of AIDS-related lymphoma [12]. HIV-positive PBL is highly aggressive with a poor prognosis; its median survival after diagnosis is only 6 months [13,14]. Nowadays, increasing reports about extraoral PBL of immunocompetent patients have emerged. Research by Castillo et al. has shown that HIV-negative PBL has different clinicopathological features compared to HIV-positive PBL. HIV-positive PBL patients are younger and more commonly exhibit oral involvement and expression of CD20, CD56, and EBER. HIV-positive PBL has a higher response to chemotherapy and a relatively longer survival [10].

HIV-negative PBL is a distinct entity which requires deep research. Because it is uncommon, we can only analyze and summarize sporadic cases. Epstein-Barr virus infection is more common in HIV-positive PBL [15]. Usually, PBL individuals without HIV infection have an immunosuppression background. A report by Teruya-Feldstein et al. demonstrated that, among six HIV negative individuals, two cases had iatrogenic immunosuppression [16]. One leg skin PBL patient had received a previous renal transplant [17], and another suffering from ulcerative colitis had been administered azathioprine [18]. In our case, negativity for EBER and the lack of an immunosuppressive condition are rather rare.

Immunophenotypically, PBL strongly expresses CD38, CD138, and MUM1, negatively expresses CD20 and PAX5, and variably expresses CD79a, CD56, CD45, CD10, CD30, and EBV-EBER [19]. In situ hybridization can detect the expression of EBER in 74% of cases [20]. It is difficult to differentiate PBL from plasmablastic plasma cell myeloma especially in cases of extraoral sites without HIV infection. In such circumstances, clinical presentation plays an important role in differential diagnosis [13]. PBL has no paraproteinemia, while the M-spike is essential for the diagnosis of plasmablastic plasma cell myeloma. Bone lytic lesions are rare in PBL except at the widely metastatic stage. However, pain or fracture caused by osteolytic bone destruction is the most common symptom for plasmablastic plasma cell myeloma [13]. Usually, PBL has a higher Ki-67 index (>85%) than plasmablastic plasma cell myeloma (5% to 60%) [13,21]. Our case has the classic immunophenotypical and clinical features of PBL. Similar to the case documented by Lin et al. [22], our case presented initially in a nodal site and had a dominant pattern of nodal involvement in an immunocompetent individual. Due to its rarity, there is no standard treatment for PBL. Current treatment strategies of PBL often mirror those from regimens for aggressive non-Hodgkin’s lymphoma [5,23]. Intensive chemotherapy followed by consolidate radiotherapy can achieve an acceptable response for early stage patients with good performance [6,15]. Saraceni et al. reported that an HIV-negative, stage IIE PBL patient attained complete remission of nearly 4 years after six cycles of chemotherapy and irradiation of 45 Gy [6]. Similarly, in a case of HIV-negative PBL in the anorectal junction, three cycles of CHOP (cyclophosphamide, doxorubicin, vincristine, and prednisone) chemotherapy and involved field irradiation brought a complete remission of nearly 5 years [15]. However, for elderly or poorly performing individuals, radiotherapy is usually initiated to rapidly relieve the discomfort. We must pay attention to side effects carefully. Thakral et al. reported on an 84-year-old female with PBL in the pelvis who suffered from bowel ischemia and infarction in the radiation field [13]. Advanced radiotherapy technologies, such as IMRT and RapidArc®, can maximize the dose to the tumor and minimize the dose to normal tissues. Thus, radiation-related toxicity can be greatly reduced. In our case, such advanced technologies were applied and the patient responded dramatically to radiotherapy with good tolerance.

Recent studies have reported that bortezomib, a proteasome inhibitor, could achieve good effects in HIV-negative PBL, especially in recurrent or resistant cases after several lines of treatment [5,23]. The primary tumor was hypersensitive to bortezomib, which might cause tumor lysis syndrome [23]. Thus, close surveillance and best care support should be given after bortezomib. Additionally, in HIV-positive PBL cases, bortezomib could also achieve a dramatic response [24].

For HIV-negative PBL, autologous stem cell transplantation could be of benefit for short-term disease-free survival (up to 2 years) [25]. Other new drugs, such as brentuximab vedotin, have been reported to achieve response in CD30-positive cases [26]. Research data showed that CD30 expression accounted for 30% of all PBL cases [27-29]. Holderness et al. revealed that in a CD30-positive, left supraclavicular PBL mass without HIV infection, brentuximab vedotin made the tumor shrink remarkably while multiple chemotherapy and irradiation had failed [26].

## Conclusions

HIV-negative PBL is a rare but distinct clinicopathological malignancy. There is no optimal treatment strategy. Our case supplemented valuable information for HIV-negative PBL in the neck. The effect of radiotherapy was verified in this case. Thus, radiotherapy might be the first option for elderly or poorly performing patients.

## Consent

Written informed consent was obtained from the patient for publication of this case report and accompanying images. A copy of the written consent is available for review by the Editor-in-Chief of this journal.

## Abbreviations

EBER: Epstein-Barr virus-encoded small RNA
HIV: Human immunodeficiency virus
IMRT: Intensity-modulated radiotherapy
PBL: Plasmablastic lymphoma
